# Comparison of Postoperative Outcomes in 71 Patients Undergoing Cataract Surgery at a Single Center with and Without Preoperative Keratostill Moisturizing Eye Drops

**DOI:** 10.3390/jcm14124349

**Published:** 2025-06-18

**Authors:** Piotr Miklaszewski, Anna Maria Gadamer, Dominika Janiszewska-Bil, Anita Lyssek-Boroń, Dariusz Dobrowolski, Edward Wylęgała, Beniamin Oskar Grabarek, Katarzyna Krysik

**Affiliations:** 1 Department of Ophthalmology, St. Barbara Hospital, Trauma Centre, 41-200 Sosnowiec, Poland; annagadamer978@gmail.com (A.M.G.); dominika.bjaniszewska@gmail.com (D.J.-B.); anitaboron3@gmail.com (A.L.-B.); dardobmd@wp.pl (D.D.); kkrysik@gmail.com (K.K.); 2 Department of Ophthalmology, Faculty of Medicine, Academy of Silesia, 40-555 Katowice, Poland; 3 Collegium Medicum, WSB University, 41-300 Dabrowa Gornicza, Poland; bgrabarek7@gmail.com; 4 Department of Ophthalmology, District Railway Hospital, 40-760 Katowice, Poland; wylegala@gmail.com; 5 Department of Ophthalmology, Faculty of Medicine, Medical University of Silesia, 40-555 Katowice, Poland

**Keywords:** cataract surgery, dry eye disease, ocular surface condition, tear film stability, ocular surface disease index (OSDI), tear break-up time (TBUT), optical coherence tomography (OCT), corneal epithelial thickness

## Abstract

**Background/Objectives**: Dry eye disease (DED) is a common condition that can significantly impact cataract surgery outcomes. Preoperative management strategies, including the use of moisturizing eye drops, may improve ocular surface health and postoperative recovery. This study aimed to compare postoperative outcomes in 71 patients undergoing cataract surgery between June 2022 and May 2023 at a single center with and without preoperative keratostill moisturizing eye drops (sterile aqueous 0.3% hydroxypropyl methylcellulose solution) determined using the ocular surface disease index (OSDI), tear break-up time (TBUT), and optical coherence tomography (OCT) at diagnosis, on the day of surgery, and at two weeks postoperatively. **Methods**: A prospective observational study was conducted on 71 patients undergoing cataract surgery at Saint Barbara Hospital Trauma Center, Sosnowiec, Poland, from June 2022 to May 2023. Patients were randomly assigned to a test group (moisturizing eye drops) or a control group (no preoperative eye drops). The OSDI, TBUT, and OCT were evaluated at the baseline, preoperatively, and postoperatively. **Results**: The test group showed a significant improvement in OSDI scores (preoperative: 6.34 vs. baseline: 11.81; *p* < 0.001), which further decreased postoperatively (3.30; *p* < 0.001). TBUT also significantly increased from baseline to the preoperative visit (6.20 s to 7.97 s; *p* = 0.002) and remained stable after surgery (7.78 s). In contrast, the control group demonstrated only a minimal postoperative change in OSDI (3.92 to 3.70; *p* > 0.05) and a significant postoperative decrease in TBUT (5.96 s to 5.69 s; *p* = 0.864). Only the control group showed a significant postoperative decrease in epithelial thickness in operated eyes (*p* = 0.021), whereas no significant changes were observed in the test group. **Conclusions**: The preoperative use of moisturizing eye drops significantly improves the tear film stability, ocular comfort, and epithelial integrity, leading to better postoperative outcomes in cataract surgery patients.

## 1. Introduction

Cataract surgery is one of the most frequently performed surgical procedures worldwide, aiming to restore vision impaired by cataracts, which is a leading cause of global blindness [1]. While typically effective, this procedure often comes with complications due to concurrent ocular surface disorders, such as dry eye disease (DED), which can significantly affect the healing process and overall patient well-being post-surgery [2,3]

DED is a significant clinical concern that can complicate both the cataract surgery procedure and the postoperative recovery period [4]. The high incidence of DED following cataract surgery may result from corneal nerve transection, leading to impaired epithelial wound healing, increased permeability, reduced epithelial metabolic activity, and a loss of cytoskeletal structures, ultimately decreasing corneal sensitivity and tear production [4]. Similarly, studies have shown that corneal nerve damage caused by surgical incisions and exposure to intense operating microscope light may contribute to a mucin deficiency, tear film insufficiency, and instability [5]. The postoperative use of antibiotic-steroid eye drops, tear film instability due to surface irregularities at the incision site, and reduced mucin production from the conjunctiva may further exacerbate DED symptoms [6]. Moreover, pre-existing DED can worsen a phenomenon observed in many patients [2,7,8,9].

The literature suggests various preoperative management strategies aimed at attempting to improve the ocular surface, such as tear substitutes and lubricating ointments, Cyclosporine 0.05%, the LipiFlow Thermal Pulsation System, hydroxypropyl guar (HPG) and hyaluronic acid (HA) ophthalmic solutions, Lifitegrast, betamethasone acetate 0.1%, and punctal plugs or punctal cautery [10,11,12,13,14]. For patients with MGD (Meibomian Gland Dysfunction), lid hygiene, warm compresses, and omega-3 fatty acid supplementation may also be recommended [8].

Recent evidence underscores the importance of the preoperative assessment and management of DED to optimize cataract surgery outcomes [9]. A systematic review by Naderi et al. highlights that untreated DED before cataract surgery can impair biometric measurements, contribute to postoperative discomfort, and delay visual recovery [9]. Their findings suggest that stabilizing the ocular surface before surgery may enhance the accuracy of intraocular lens (IOL) calculations and improve overall patient satisfaction [9]. Despite these recommendations, there remains a lack of standardized protocols for preoperative DED management, necessitating further research to establish clear clinical guidelines [9].

Ensuring appropriate patient preparation for the procedure, especially for those with pre-existing DED, is, therefore, an integral part of the treatment process aimed at maximizing the effectiveness of the surgery and post-operative patient comfort [8].

The Ocular Surface Disease Index (OSDI) is a validated and widely used questionnaire designed to quantify the subjective symptoms of DED, including discomfort, visual disturbance, and the impact on daily functioning. Its effectiveness was validated in a study that showed the OSDI, along with the Symptom Assessment in Dry Eye (SANDE), had the highest ability to distinguish between DED patients and healthy individuals compared to other questionnaires, including the 5-Item Dry Eye Questionnaire (DEQ-5), McMonnies Dry Eye Questionnaire, and Standard Patient Evaluation of Eye Dryness (SPEED) [15].

Additionally, an analysis published in the Indian Journal of Ophthalmology (2021) found that the OSDI correlated with all diagnostic tests for DED, further supporting its broad application in assessing the disease severity [16]. In contrast, DEQ-5 showed a significant correlation only with the tear film breakup time (TBUT) and corneal staining [16]. This indicates that the OSDI provides a more comprehensive evaluation of dry eye symptoms and their impact on patients’ daily lives [16].

Due to its strong correlation with clinical signs and its ability to reflect the DED severity across multiple domains, the OSDI remains a core diagnostic tool for assessing patient-reported symptoms and monitoring treatment outcomes in both clinical and research settings [16].

The TBUT test is a commonly used method for assessing tear film stability in diagnosing DED [17]. It involves applying a drop of fluorescein dye to the eye and measuring the time from the last blink until the tear film breaks up [17]. TBUT values below 10 s indicate abnormalities in the tear film, suggesting the presence of dry eye [17]. Analyses conducted by Paugh and colleagues confirmed the usefulness of the fluorescein tear break-up time test as an important diagnostic tool for dry eye, applicable in cases of both Meibomian Gland Dysfunction (MGD) and an aqueous tear deficiency [17].

Using the OSDI and TBUT together provides a comprehensive approach to assessing the ocular surface condition [18]. A study evaluating their relationship in computer users found that higher OSDI scores, indicating more severe symptoms, were associated with a shorter TBUT, reflecting decreased tear film stability [18]. Additionally, research on the impact of dry eye disease on daily functioning showed that a worse TBUT and higher OSDI scores were linked to greater work impairment and reduced activity levels [18]. These findings suggest that combining the OSDI and TBUT not only improves the accuracy of dry eye disease diagnosis and assessment, but also provides insight into its functional impact on patients’ daily lives [18,19].

Optical Coherence Tomography (OCT) is a non-invasive imaging technique that enables the high-resolution cross-sectional visualization of the ocular surface, allowing for the precise assessment of corneal epithelial thickness and overall tear film stability [20]. OCT plays a crucial role in the diagnosis and monitoring of DED, as structural changes in the corneal epithelium are often associated with ocular surface dysfunction [20]. This study demonstrated that the application of OCT in corneal epithelial thickness mapping can improve the accuracy of DED diagnosis [20]. The analysis of epithelial profiles in DED patients and the control group revealed significant differences in the corneal epithelial thickness, suggesting that OCT mapping may be a valuable tool for identifying this disease [20].

Similarly, a study demonstrated that patients with DED have a highly irregular corneal epithelial surface compared to healthy individuals [21]. It was observed that epithelial thickness variability was strongly correlated with the severity of DED symptoms, highlighting the potential of OCT in monitoring disease progression and treatment efficacy [21].

Therefore, this study aimed to compare postoperative outcomes in 71 patients undergoing cataract surgery between June 2022 and May 2023 at a single center with and without preoperative keratostill moisturizing eye drops (sterile aqueous 0.3% hydroxypropylmethylcellulose solution (HPMC)) determined using the OSDI, TBUT, and OCT at diagnosis, on the day of surgery, and at two weeks postoperatively.

## 2. Materials and Methods

### 2.1. Ethics

This prospective observational study with intervention was conducted with the approval of the Ethics Committee (Nr 24/KB/AŚ/04/2024), obtained on 3 April 2024, and in accordance with the principles of the Declaration of Helsinki. Written informed consent was obtained from all participants.

### 2.2. Study Design

This study was conducted on a group of 71 patients undergoing cataract surgery at the Ophthalmology Department of Saint Barbara Hospital, Trauma Center, Sosnowiec, Poland, between June 2022 and May 2023. All participants were diagnosed with age-related cataracts based on slit-lamp biomicroscopy, best-corrected visual acuity (BCVA) assessment, and lens opacity grading.

The inclusion criteria encompassed patients with visually significant cataracts causing functional visual impairment, defined by BCVA worse than 0.6 (decimal scale), with symptoms such as glare or reduced contrast sensitivity. Cataract density was graded using the Lens Opacities Classification System III (LOCS III) and included grades NO2 to NO4. Patients were qualified for surgery based on these criteria, independent of group allocation. The average phacoemulsification procedure time was 12 ± 3 min.

All surgeries were performed by a single experienced surgeon (P.M.) using a standardized phacoemulsification technique (stop-and-chop method) under topical anesthesia. The procedure involved a clear corneal incision, continuous curvilinear capsulorhexis, nucleus hydrodissection, fragmentation and phacoemulsification, cortical aspiration, and in-the-bag intraocular lens (IOL) implantation, followed by incision sealing. The average duration of surgery was approximately 12 ± 3 min. All procedures were uneventful, with no intraoperative complications reported.

Patients were categorized into two groups based on preoperative intervention. The test group, consisting of 37 patients, received Keratostill moisturizing eye drops containing 0.3% HPMC and dexpanthenol. These eye drops, manufactured by Bruschettini s.r.l., Genova, Italy, and distributed in Poland by Pharm Supply Sp. z o.o. (Warszawa, Poland), were administered as one drop per eye, five times daily, for seven days before cataract surgery. The control group, comprising 34 patients, did not receive preoperative moisturizing eye drops.

The primary outcomes of this study were aimed at assessing the impact of preoperative moisturizing eye drops on key clinical parameters related to dry eye disease in cataract surgery patients. These outcomes included OSDI and TBUT. Secondary outcomes were intended to provide additional insights into the effects of the intervention and explore potential predictors of response to treatment. These outcomes included corneal epithelial thickness, measured via OCT, which was used to evaluate the integrity of the corneal surface. The secondary outcome was the change in epithelial thickness at the central corneal zone from baseline to the postoperative period. Stability or improvement in epithelial thickness in the test group compared to the control group was considered a positive outcome. Secondary outcomes also included the exploration of gender-based differences in response to preoperative eye drop treatment.

#### Cataract Staging

The severity of cataracts was graded using the Lens Opacities Classification System III (LOCS III) based on nuclear color and opalescence. Patients included in this study had grades ranging from NC2–NC4, with the majority (68%) presenting with moderate nuclear opalescence (NC3). This ensured relative homogeneity of lens density across the study cohort.

### 2.3. Participant Exclusion Criteria

All patients underwent complete ophthalmic examination including best corrected-distance visual acuity, IOP measurement by air puff tonometry (Goldman tonometry was not used so as not to damage the corneal surface), slit-lamp biomicroscopy, and fundus examination with a dilated pupil. The TBUT test and OCT examination were performed before the use of mydriatic drops. Importantly, all preoperative measurements (TBUT, OCT-based epithelial mapping, and OSDI) were performed before instillation of any mydriatic or anesthetic agents to ensure accurate baseline assessment of tear film and ocular surface status.

### 2.4. Group Assignment and Randomization

All patients included in this study were diagnosed with age-related cataract and subsequently underwent cataract surgery. Eligibility criteria required patients to be candidates for cataract surgery, while those with dry eye disease or other ocular or systemic conditions that could influence corneal epithelium thickness measurements were excluded. Exclusion criteria encompassed corneal pathologies (including dystrophies, degenerations, scars, and neovascularization) and previous ocular surgeries or trauma, glaucoma, uveitis, and systemic diseases with ocular manifestations (such as diabetes, connective tissue disorders, and skin or mucous membrane diseases). Additionally, patients using any topical medication, particularly those containing preservatives, were not included.

Patient allocation to the test and control groups was conducted through randomization, with every other eligible patient being assigned to receive moisturizing eye drops preoperatively. The observed difference in baseline OSDI values between the groups was attributed to random variation rather than systematic bias in the assignment process. The implementation of randomization aimed to minimize bias and the comparable baseline TBUT values further supported the overall equivalence of the groups.

### 2.5. Schedule

All measurements, including TBUT, epithelial thickness, and the OSDI questionnaire, were conducted by a single researcher. The study parameters encompassed TBUT, the OSDI test, and epithelial mapping in the central corneal zone, with assessments performed at three distinct time points: (1) during the preoperative qualification visit (2–8 weeks before surgery), (2) on the day of surgery, and (3) two weeks postoperatively during the follow-up visit.

The screening visit (first visit) occurred 2–8 weeks before surgery, during which patients were evaluated, and these results were considered baseline values. During this period, patients in the test group were instructed to use moisturizing eye drops for one week prior to surgery. On the day of surgery (second visit), immediately before the procedure, additional measurements were obtained to assess the impact of the one-week eye drop intervention on the ocular surface, and these results were compared with baseline values. The final measurements (third visit) were conducted two weeks postoperatively during a follow-up visit to evaluate the combined effects of both the preoperative eye drop intervention and surgery on the ocular surface.

The selection of a two-week observation period aligns with standard clinical practice at our center, where the second follow-up visit after cataract surgery typically takes place at this interval. By this time, the initial healing process is generally complete, allowing for a reliable assessment of early surgical outcomes and the efficacy of interventions such as moisturizing eye drops.

Measurements were obtained from patients using and not using artificial tears before surgery. Patients in the test group applied 0.3% HPMC solution (Keratostill; Bruschettini s.r.l., Italy) five times daily for seven days preoperatively, while those in the control group did not receive artificial tears before surgery. To avoid introducing additional confounding variables, 0.3% HPMC solution was not administered postoperatively in the test group. Additionally, perioperative corticosteroids (dexamethasone) were used as part of standard postoperative management to reduce the risk of inflammatory complications following surgery.

#### Measurement Tools and OCT Parameters

Corneal parameters were measured using Spectral Domain Optical Coherence Tomography (SOLIX, Optovue, Fremont, CA, USA). Optovue SOLIX is an advanced spectral-domain optical coherence tomography (SD-OCT) system developed by Visionix (Pont-de-l’Arche, France), offering high-quality imaging of ocular structures. It operates at a speed of 120,000 A-scans per second, enabling the acquisition of images with very high resolution. The device provides a full range of scanning for both anterior and posterior eye structures. SOLIX particularly excels in imaging and measuring the thickness of the corneal epithelium. Precise mapping of epithelial thickness is crucial for detecting and monitoring conditions such as keratoconus, dry eye, and post-operative assessments after refractive surgeries. Advanced software like AngioVue software (version 2017.1.0.155; Optovue Inc., Fremont, CA, USA) in data analysis, providing comprehensive reports on epithelial thickness and other corneal layers, allowing for a better understanding of corneal health and planning appropriate medical interventions. Additionally, the device features an integrated database, enabling comparison of results with healthy norms, thus enhancing diagnostic accuracy.

### 2.6. OSDI Questionnaire

OSDI is a widely used, standardized patient-reported questionnaire designed to assess symptoms of DED and its impact on daily life. It provides a quantitative measure of ocular discomfort, vision-related function, and environmental triggers associated with dry eye syndrome.

The OSDI questionnaire consists of 12 questions, categorized into three domains: (1) ocular symptoms (e.g., dry eyes, grittiness, pain, and blurred vision), (2) visual function (e.g., difficulty reading, watching TV, or using digital screens), and (3) environmental factors (e.g., discomfort in windy or air-conditioned environments). Patients rate each symptom on a scale from 0 (no symptoms) to 4 (severe symptoms), and the final OSDI score is calculated to classify the severity of dry eye disease, with higher scores indicating more severe ocular surface dysfunction. The OSDI was administered at three time points: during the baseline visit (2–8 weeks before surgery), on the day of the surgery (preoperative assessment), and at the follow-up visit two weeks postoperatively. Higher OSDI scores indicate greater symptom severity, with established cutoffs defining mild, moderate, and severe dry eye disease.

#### Modification of the OSDI Questionnaire

Due to the fact that all patients enrolled in this study had cataract-related visual impairment, we removed two items from the standard OSDI questionnaire: “blurred vision” and “poor vision”. These symptoms were expected in nearly all participants due to lens opacity and not necessarily reflective of dry eye disease. The removal aimed to avoid inflating the OSDI score with cataract-related symptoms and is supported by prior studies, including Kasetsuwan et al. [22]. We acknowledge that the OSDI is a validated tool and that any modification may affect its psychometric properties. In this context, the modified score should be interpreted with caution, and our findings are limited by the absence of formal revalidation. Nevertheless, the internal consistency and responsiveness of the modified OSDI were acceptable over time. Future research may consider validating an adapted version suitable for patients with visually significant cataracts.

### 2.7. TBUT Questionnaire

A sterile fluorescein strip moistened with saline was applied to the lower conjunctival sac of each eye. Patients were asked to blink naturally to distribute the dye across the ocular surface. Under a slit-lamp biomicroscope equipped with a cobalt blue filter, the examiner recorded the time from the last blink until the first visible break or dark spot appeared in the tear film, indicating tear instability. TBUT was measured three times per eye and the average value was used for analysis. A TBUT value of ≥10 s was considered normal, whereas values between 5 and 9 s suggested borderline tear film instability, and values <5 s indicated significant tear film dysfunction. The test was conducted at the same three time points as the OSDI assessment to evaluate changes in tear film stability over the course of the study.

### 2.8. Statistical Environment

Analyses were conducted using the R Statistical language (version 4.3.1) on Windows 10 pro 64 bit (build 19045), using the packages rmcorr (version 0.6.0), effectsize (version 0.8.6), rio (version 1.0.1), robustlmm (version 3.2.3), emmeans (version 1.8.9), parameters (version 0.21.2), insight (version 0.19.6), report (version 0.5.7), gtsummary (version 1.7.2), readxl (version 1.4.3), and dplyr (version 1.1.3 and psych (version 2.3.9).

The level of statistical significance was established at α = 0.05. For analyzing differences between two independent groups for numerical variables with non-normal distributions, the Wilcoxon rank sum test was the chosen method. The independence of two categorical variables was determined using the Pearson chi-squared test and Fisher’s exact test.

#### 2.8.1. Robust Linear Mixed-Effects Model (RLMM)

To explore the effects of potential predictors of outcomes related to Dry Eye Syndrome, as measured by the OSDI, TBUT test, and OCT, we employed a comprehensive statistical approach using the RLMM. The RLMM, based on the “random effects contamination model,” allowed for the effective management of multiple sources of data contamination without necessitating superfluous assumptions about the data’s grouping structure. The solitary assumption was that the model parameters were estimable, a requirement that was equally essential for both classical and robust estimation approaches [23].

This model’s strength resided in its capacity to address potential outliers or contamination across various levels of random variation present in our dataset. This functionality was particularly crucial in our study context, given the diverse sources of measurements including different time points, distinct groups, and both operated and non-operated eyes. To operationalize this robust analytical methodology, we employed the Robust Scoring Equations estimator, as formulated by Koller [24], designed to fit linear mixed-effects models robustly, effectively managing outliers and contamination. To account for potential outliers or non-normality in the data and improve the robustness of the model estimates, the RLMM model utilized smoothing techniques known as smoothed Huber functions.

Our RLMMs incorporated both fixed and random effects, thereby addressing both independent and clustered variability in the data. The fixed-effects component included several explanatory variables: eye status (operated vs. non-operated), time (baseline, preoperative, and postoperative), and group (control vs. test). To manage potential confounding, the models also included age and gender variables, which independently influenced our outcomes of interest.

#### 2.8.2. Correlation Analyses

Repeated measures correlation coefficients were used to estimate the relationships between numerical variables in the context of repeated measurements. The use of this coefficient was justified by the specific structure of our data, in which the same variables were measured repeatedly in the same study participants. Traditional correlation methods might not account for within-subject correlations, which could lead to incorrect conclusions. The repeated measures correlation coefficient allows for precise estimation of the strength of the relationship between variables by eliminating the effect of individual differences, which is crucial for accurate data analysis in a project involving repeated measurements. The applied methodology was based on some of the works [25,26,27].

#### 2.8.3. Sample Size Analysis

The sample size determination for this study was based on power calculations designed to ensure that this study could detect clinically meaningful differences in OSDI and TBUT between the test and control groups. The anticipated effect size was based on previous studies examining the impact of moisturizing eye drops on OSDI and TBUT scores in patients undergoing cataract surgery. From the literature, a medium effect size of approximately 0.5 was considered reasonable for detecting differences between groups, as the intervention was expected to result in moderate improvements in ocular surface parameters. A statistical power of 80% (β = 0.20) was chosen to minimize the risk of Type II errors (failing to detect a difference when one exists). The significance level (α = 0.05) was used to control the probability of Type I errors (detecting a difference when none exists). Using a standard formula for comparing means between two independent groups (test vs. control), the following Equation was applied:

n = (Zα/2 + Zβd)2·2σ2n = (dZα/2 + Zβ)2·2σ2

where:

Zα/2 = 1.96Zα/2 = 1.96 for a two-tailed test at α = 0.05.
Zβ = 0.84Zβ = 0.84 for 80% power.
d = Effect Size = 0.5d = Effect Size = 0.5.
σσ = standard deviation from similar studies.

Based on this calculation, a minimum of 64 participants was deemed necessary to detect a medium effect size with 80% power. However, to account for potential dropouts and incomplete data, the sample size was increased by 10%, leading to a final sample size of 71 participants.

## 3. Results

### 3.1. Patient Demographics and Baseline Characteristics of Study Groups

This study involved 71 patients who underwent cataract surgery, bifurcated equitably into control (47.9%, n = 34) and test groups (52.1%, n = 37). Females accounted for the majority of the sample, representing 62.0% (n = 44), while males constituted the remaining 38.0% (n = 27). The age distribution showcased a median of 72.0 years, with a consistent IQR from 69.0 to 76.5 years, indicating a sample largely comprised of older adults. The gender and age distributions exhibited no significant variance across the control and test groups (*p* = 0.152 and *p* = 0.995, respectively). This demographic parity enhances the study’s validity, bolstering confidence in the conclusions drawn regarding the intervention’s impact. Table 1 presents the demographic data of subjects.

Preoperative BCVA in the study population averaged 0.43 ± 0.12 (decimal scale), which improved significantly to 0.78 ± 0.10 at two weeks postoperatively (*p* < 0.001), reflecting effective surgical outcomes. The cataract density, assessed using LOCS III, showed an even distribution among included patients, with most falling between the NO2 and NO4 categories.

### 3.2. Baseline Medical Parameters: OSDI, TBUT, and Corneal Epithelial Thickness

Although the full TFOS DEWS II diagnostic battery was not used, our combined use of a symptom-based (OSDI), a tear stability (TBUT), and a structural marker (epithelial thickness) approach provided a pragmatic and clinically meaningful assessment of the dry eye status. This strategy is supported by several prior studies in cataract surgery settings. Except for the OSDI, all parameters were separately scrutinized for both the operated and non-operated eyes. The key differentiation between the control and test groups was the usage of eye drops between the baseline and surgical stages. Table 2 exhibits the distribution of the medical parameters at the baseline for both groups.

From the baseline data presented in Table 2, no significant differences were detected in either the TBUT or corneal thickness across various corneal points. The only parameter that presented a notable difference was the OSDI, with the test group exhibiting higher scores, indicative of elevated ocular discomfort or visual impairment within this group.

### 3.3. Gender-Based Variability in OSDI Improvement Following Eye Drop Use

Among female participants (n = 25), a substantial majority (84.0%, n = 21) reported an improvement in their eye condition following the use of the drops. Conversely, only a small fraction (16.0%, n = 4) reported no change in their condition. This suggested that the eye drops were largely effective in improving ocular symptoms among female participants.

The perceived efficacy of the eye drops among male participants (n = 12), however, was less pronounced. A smaller majority (58.3%, n = 7) reported an improvement in their eye condition, while a notable proportion (41.7%, n = 5) observed no change. This indicated a reduced perceived effectiveness of the eye drops in the male subset of the test group. While not statistically significant (*p* = 0.116), the observed differences could potentially have clinical implications. These observations suggested a potential gender-based difference in response to the eye drops. This could be attributed to a variety of factors, including inherent biological differences, differential adherence to treatment, varying subjective perceptions of symptom improvement, or even differences in the baseline severity of the eye condition.

#### 3.3.1. Overview of OSDI Analysis

Our research utilized the RLMM to explore the effects of age, gender, and the interaction between the group and time on the OSDI score while accounting for intra-patient variability. The model, which omitted the “status eye” factor from the interaction component, explained a substantial proportion of the total variation in the data, with marginal and conditional R-squared values of 0.77 and 0.20, respectively. The random effects component of the model revealed significant variability across patients, as indicated by a standard deviation of 5.82. This underscores the importance of accounting for individual differences in disease manifestation and progression, reinforcing the inclusion of patients as a random effect in our model.

#### 3.3.2. Effect of Age and Gender on OSDI

The analysis of fixed effects demonstrated that age did not have a statistically significant impact on the OSDI scores (*p* = 0.751). This suggests that once other variables in the model were accounted for, age did not significantly alter the OSDI score. Consequently, our data did not support age as a noteworthy determinant of ocular surface disease severity. Conversely, gender emerged as a significant predictor of OSDI scores (*p* = 0.038). On average, male patients reported OSDI scores that were approximately 3.15 units lower than those of their female counterparts, assuming all other variables remained constant. This finding highlights a discernible gender disparity in the OSDI scores, suggesting that males in our dataset tended to experience less severe symptoms of ocular surface disease.

#### 3.3.3. OSDI Changes over Time—Estimated Marginal Means (EMMS) Analysis

To thoroughly assess the effect of the interaction between the group and time on the OSDI scores, an analysis based on EMMS was conducted for different groups and time points, as presented in Table 3. During the Baseline Phase, the test group exhibited the highest mean OSDI score (EMM = 11.81) compared to the control group (EMM = 3.92), indicating more severe ocular surface disease symptoms. In the Preoperative Phase, the test group’s mean OSDI score decreased to 6.34, while the control group showed a slight increase to 4.88, suggesting that the use of eye drops contributed to symptom improvement in the test group. Postoperatively, both groups experienced a decrease in the mean OSDI scores, with the test group reaching an EMM of 3.30 and the control group 3.70, demonstrating the potential effectiveness of the combined intervention of eye drops and surgery in alleviating symptoms.

#### 3.3.4. OSDI Contrast Analysis—Differences over Time Between Groups

A contrast analysis of different time points within each group, as presented in Table 4, provided a more detailed understanding of the interaction effect. In the control group, no significant differences were observed across the various time points (all *p* > 0.05), indicating that the condition remained relatively stable throughout the study period. In contrast, the test group exhibited significant differences at all time points (all *p* < 0.001), with the largest effect size observed between the baseline and postoperative phases (d = 1.46), suggesting a substantial improvement following the intervention. Additionally, the significant decrease in OSDI scores between the preoperative and postoperative phases (d = 0.52) further confirmed the positive effect of the intervention.

#### 3.3.5. Comparative Statistical Analysis of OSDI Across Groups and Time Point

Our research also examined the effects of age, gender, and the interaction of the eye status, group, and time on the OSDI, with the model explaining a substantial proportion of the total variation (conditional R^2^ = 0.41, marginal R^2^ = 0.09). A detailed contrast analysis of the OSDI between the control and test groups at distinct time points is presented in Table 5. In the Baseline Comparison, a significant difference in OSDI scores was observed between the groups (estimate = −7.89, *p* < 0.001, d = −1.36), with the test group showing substantially higher scores, indicating more severe ocular surface disease. During the Preoperative Phase, the difference in the OSDI scores between the groups decreased considerably (estimate = −1.47) and was not statistically significant (*p* = 0.350), suggesting that both groups exhibited more similar conditions, likely due to natural disease fluctuations and the effects of preoperative eye drops. In the Postoperative Phase, the test group’s OSDI scores slightly exceeded those of the control group (estimate = 0.40), but the difference was not statistically significant (*p* = 0.799). This indicated that the intervention, including preoperative moisturizing eye drops and post-operative anti-inflammatory medications, effectively improved the test group’s ocular surface condition, bringing their OSDI scores in line with the control group.

### 3.4. Overview of TBUT Analysis

Our research utilized the RLMM to explore the effects of age, gender, and the interaction of the eye status, group, and time on the TBUT while accounting for intra-patient variability. The model explained a substantial proportion of the total variation in the data, with conditional and marginal R-squared values of 0.41 and 0.09, respectively.

#### 3.4.1. Effect of Age and Gender on TBUT

The coefficient for age was negative (B1 = −0.03), suggesting a slight decline in the TBUT with increasing age, though this effect was not statistically significant (*p* = 0.189). This implied that while there was a tendency for the TBUT to decrease with age, this trend was not strong enough to be definitive within the context of this study. The coefficient for gender (B2 = −0.80) indicated that the TBUT for males was lower than for females by approximately 0.80 s, with this effect approaching significance (*p* = 0.079), although this result warranted further investigation given the borderline *p*-value.

#### 3.4.2. TBUT Changes in Non-Operated Eyes

At the baseline, the EMM of the TBUT was slightly higher in the control group (6.55) compared to the test group (6.40), though this difference was not substantial. In the Preoperative Phase, the control group experienced a slight decrease in the TBUT (EMM = 6.34), while the test group showed an increase (EMM = 7.15). By the Postoperative Phase, both groups exhibited an overall increase in the TBUT, with the test group (EMM = 7.12) slightly lower than the control group (EMM = 7.14). These findings indicate that both groups demonstrated comparable TBUT responses throughout this study, suggesting that the intervention may have positively influenced the tear film stability in both groups.

#### 3.4.3. TBUT Changes in Operated Eyes

At the baseline, the EMM of the TBUT was higher in the control group (6.78) compared to the test group (6.20). During the Preoperative Phase, the control group experienced a decrease in the TBUT (EMM = 5.96), whereas the test group exhibited a substantial increase (EMM = 7.97), likely due to the effect of using eye drops. In the Postoperative Phase, the TBUT further declined for the control group (EMM = 5.69), while it slightly decreased for the test group (EMM = 7.78). These findings indicate that the intervention had a more beneficial effect on the operated eyes in the test group.

While no significant changes in the TBUT were observed in both the non-operated and operated eyes within the control group, the test group demonstrated a significant increase in the TBUT in the operated eye from the baseline to the preoperative phase (+1.76 s). This suggests that the use of eye drops was particularly effective for patients in the test group who underwent surgery. Notably, even after discontinuing the drops post-cataract surgery, the improvement in the TBUT remained relatively stable (+1.58 s from the baseline to three weeks after surgery). Furthermore, the larger Cohen’s d values indicated a substantial effect size, reinforcing the positive impact of the intervention on tear film stability in the test group.

#### 3.4.4. Comparative Analysis of TBUT Across Study Groups

At the baseline, no significant disparities in the TBUT were found between the control and test groups, regardless of whether the eyes were non-operated or operated. This conclusion was supported by the modest effect sizes indicated by Cohen’s d values, emphasizing the minimal influence of these initial differences. During the preoperative phase, the TBUT values did not significantly differ between control non-operated, test non-operated, and control operated eyes. However, a significant increase in the TBUT was observed in test operated eyes compared to control operated eyes (*p* = 0.011), with a large effect size (Cohen’s d = −1.27), indicating a considerable improvement in tear film stability in the test operated group. In the postoperative phase, eye drops appeared to not only balance, but even enhance the TBUT in the eye undergoing surgery compared to the healthy eye, with an overall shift of approximately 1.0 s (from −0.20 s at the baseline favoring the non-operated eye to 0.81 s preoperatively favoring the eye with a cataract). At the postoperative time point, no significant differences were found in the TBUT between the control non-operated and test non-operated groups. However, a significant decline in the TBUT was noted in the control operated eyes relative to the control non-operated eyes (*p* = 0.032), suggesting a reduction in the tear film stability postoperatively. Importantly, the TBUT in the test operated eyes remained significantly higher than in the control operated eyes (*p* = 0.007), even three weeks after discontinuing eye drops. Toward the end of the observation period, the difference in the TBUT between groups was approximately 2 s in the operated eyes. A detailed contrast analysis of the TBUT across different time points within each group is provided in Table 6, illustrating the statistical significance of the observed changes in both non-operated and operated eyes.

### 3.5. Overview of Corneal Epithelial Thickness Analysis

Our research utilized the RLMM to analyze the effects of various factors—age, gender, the interaction of the eye status, group, and time—at the middle control point of the corneal epithelial thickness. The model explained a significant portion of the total variation in the data, as evidenced by the conditional R-squared value of 0.77. However, the marginal R-squared value of 0.09 suggested that the fixed effects alone account for a relatively small proportion of the variance.

#### 3.5.1. Effect of Age and Gender on Corneal Epithelial Thickness

The analysis revealed that age had a slight but non-significant effect on the corneal epithelial thickness, with an estimated coefficient of B_1_ = −0.08 μm (95% CI: [−0.19, 0.03], *p* = 0.146), suggesting a minor decrease in thickness with increasing age. Similarly, the gender coefficient for males (B_2_ = 1.71 μm, 95% CI: [−0.18, 3.61], *p* = 0.076) indicated that, on average, males had a slightly thicker corneal epithelium compared to females; however, this effect was not statistically significant at the 0.05 level. The standard deviation of the intercepts among patients was estimated at 3.72 μm, reflecting variability in the baseline corneal epithelial thickness. Additionally, the residual standard deviation of 2.17 μm highlighted an unexplained variability in the thickness beyond the fixed and random effects included in the model.

#### 3.5.2. Changes in Corneal Epithelial Thickness over Time in Non-Operative Eyes

In the control group, non-operated eyes exhibited a subtle upward trajectory in their corneal epithelial thickness from the baseline to the postoperative phase, which may reflect a natural progression or the influence of an unspecified external factor. In contrast, the test group showed an initial increase in thickness from the baseline to the preoperative phase, followed by a slight decrease postoperatively. This trajectory suggests that surgery on the fellow eye may have induced a transient change in the corneal epithelial thickness, though the effect appeared to diminish over time.

#### 3.5.3. Changes in Corneal Epithelial Thickness over Time in Operative Eyes

Regardless of the group, a significant decrease in the corneal epithelial thickness was observed from the baseline to the postoperative phase, suggesting that the surgical process itself may influence the corneal epithelial thickness. The use of eye drops in the operated eyes of the test group did not lead to a significant improvement in the epithelial thickness, showing only a minimal change of −0.08 μm. In contrast, the control group’s non-operated eyes exhibited an increase in thickness of approximately 1.00 μm over the same period, despite the absence of eye drops. Across all time points, the control group consistently demonstrated a slightly higher corneal epithelial thickness than the test group, regardless of the operative status of the eyes. This trend suggests that the intervention associated with the test group may have contributed to a slight thinning of the corneal epithelium.

### 3.6. Contrast Analysis—Differences over Time Between Groups

The contrast analysis results, presented in Table 7, highlight patterns in corneal epithelial thickness over time across different eye statuses and groups. In the control group’s non-operated eyes, there was a slight, non-significant increase in thickness from the baseline to preoperative phase (est. = −0.05, *p* = 0.994), followed by a more noticeable but still non-significant increase from the baseline to postoperative phase (est. = −0.95, *p* = 0.182). Similarly, in the test group’s non-operated eyes, an increase in thickness was observed from the baseline to preoperative phase (est. = −0.93, *p* = 0.169) and from the baseline to postoperative phase (est. = −0.65, *p* = 0.419), though neither change reached statistical significance.

In the control group’s operated eyes, a non-significant decrease in epithelial thickness was observed from the baseline to the preoperative phase (Est. = 0.56, *p* = 0.555), whereas a significant reduction occurred from the baseline to the postoperative phase (Est. = 1.45, *p* = 0.021), indicating a notable thinning effect following surgery. In contrast, the test group’s operated eyes demonstrated non-significant changes in epithelial thickness across all time intervals: baseline to preoperative (Est. = 0.08, *p* = 0.987), baseline to postoperative (Est. = 0.71, *p* = 0.354), and preoperative to postoperative (Est. = 0.63, *p* = 0.442). These results suggest that while some trends in epithelial thinning were noted—particularly in the control group after surgery—most changes did not reach statistical significance.

**Figure 1:**
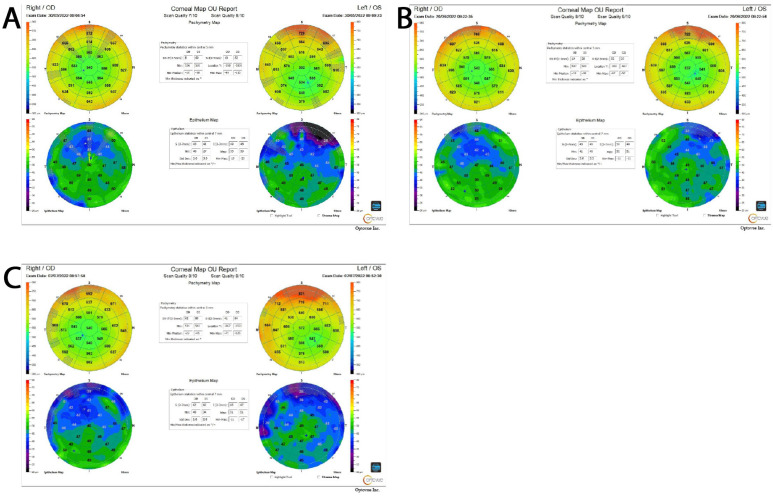
Representative OCT images from a test group at three different time points. (**A**) Screening visit (1st visit); (**B**) on the day of the surgery (2nd visit); (**C**) final measurements (3rd visit).

**Figure 2:**
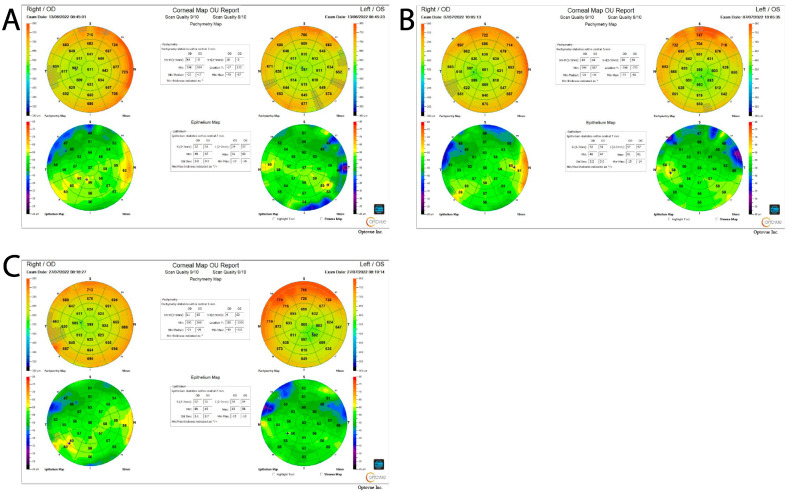
Representative OCT images from a control group at three different time points. (**A**) Screening visit (1st visit); (**B**) on the day of the surgery (2nd visit); (**C**) final measurements (3rd visit).

Differences in the corneal epithelial thickness between control non-operated and test non-operated eyes, as well as between test non-operated and test operated eyes, were not statistically significant (*p* = 0.646 and *p* = 0.341, respectively). Similarly, the thickness differences between control non-operated and control operated eyes, as well as between control operated and test operated eyes, did not reach statistical significance (*p* = 0.346 and *p* = 0.627, respectively). At the preoperative stage, none of the contrasts in the corneal epithelial thickness reached statistical significance, indicating that any changes induced by the initial stages of the intervention were not substantial enough to meaningfully affect the corneal epithelial thickness. In the postoperative period, no significant differences were observed when comparing control non-operated eyes to test non-operated eyes or test non-operated eyes to test operated eyes (*p* = 0.466 and *p* = 0.769, respectively). Likewise, the comparison between control operated and test operated eyes did not yield a statistically significant difference (*p* = 0.957). A significant increase in the corneal epithelial thickness was observed in control-operated eyes compared to control non-operated eyes (*p* = 0.028), although the effect size was small. To further illustrate changes in the corneal epithelial thickness across different time points, representative OCT images from one patient in the test group and one in the control group are provided in Figure 1 and Figure 2. These images, taken at the baseline, preoperative, and postoperative stages, visually demonstrate the trends observed in the quantitative analysis. The presented exemplary OCT images illustrate changes in the central corneal epithelial thickness in the operated eye for two patients—one from the test group who used eye drops before surgery (Figure 1) and one from the control group who did not (Figure 2).

In the test group patient, the central epithelial thickness was 46 µm before surgery, 47 µm on the day of the procedure, and 46 µm after surgery, indicating relatively stable values over the observation period. In the control group patient, the central epithelial thickness gradually decreased from 57 µm before surgery to 55 µm on the day of the procedure and 53 µm after surgery.

It is worth noting that no significant changes in the central epithelial thickness were observed in the non-operated eye, suggesting that the observed differences pertain exclusively to the surgically treated eye.

## 4. Discussion

This study aimed to investigate the impact of preoperative prophylaxis using eye drops on the ocular surface condition before cataract surgery and its subsequent effect on healing and patient well-being [28,29,30,31]. This study demonstrated that patients in the test group who received moisturizing eye drops before cataract surgery showed significant improvement in their OSDI scores and TBUT compared to the control group, suggesting better tear film stability and a reduction in dry eye syndrome symptoms [28,29,30,31]. These results are consistent with previous studies indicating an exacerbation of DED.

These findings align with the recommendations of Naderi et al., who emphasize that the preoperative management of DED can improve surgical accuracy and reduce postoperative symptoms [9]. Their review highlights that stabilizing the ocular surface before cataract surgery enhances the reliability of corneal biometric measurements, leading to more predictable refractive outcomes and greater patient satisfaction [9]. Our results support this approach, showing that the use of preoperative eye drops positively influenced the tear film stability and dry eye symptoms, as indicated by improved OSDI scores [9].

The improvement in OSDI scores suggests that the preoperative use of eye drops and postoperative anti-inflammatory treatment had a beneficial impact on the test group [32].

The higher OSDI score in the test group at the beginning of this study reflected a poorer condition of the ocular surface [9]. The decrease in this score after surgery suggests that the intervention (the use of preoperative drops) had a beneficial effect on stabilizing the tear film and reducing DED [9]. The stable OSDI scores in the control group indicate no changes in the condition of the ocular surface, which is consistent with expectations given the lack of preoperative intervention [9].

Regarding the TBUT, our study did not show a consistent significant change at all measurement points, though an improvement trend in the TBUT was observed in the test group after using the eye drops [9]. This trend suggests that preoperative intervention may contribute to better ocular surface stability, a factor also emphasized in systematic reviews as crucial for improving cataract surgery outcomes [9].

This study observed that in the test group using moisturizing drops before cataract surgery, the decrease in the corneal epithelial thickness was less than that in the control group. Although these differences were not statistically significant, this trend suggests that moisturizing drops may help maintain the stability of the corneal epithelium. The findings of Yusufoğlu and Keser indicate significant benefits from using artificial tears for the quicker stabilization of the epithelial thickness after cataract surgery, improving ocular surface regeneration and patient comfort [33]. Although our study did not show statistically significant differences in the corneal epithelial thickness between the groups, this trend is consistent with the literature and highlights the potential benefits of using artificial tears to prepare the ocular surface before surgery [33].

Importantly, the tolerance for the moisturizing drops was very good, and most patients reported subjective improvement in their eye condition after their use. In the test group, 84.0% of women and 58.3% of men felt a subjective improvement, while, respectively, 16.0% of women and 41.7% of men noticed no changes, either positive or negative, regarding their eyes and vision after the application of the drops. Significantly, no one reported a worsening of their eye condition after using the drops.

Currently, there are no strictly established guidelines for dosing these preparations. Studies, such as Pinto-Bonilla et al., have shown that patients often miss the recommended doses, which can affect the efficacy of the therapy [34]. To minimize this risk, we opted for a higher dosing frequency—5 times daily for 7 days. This regimen ensures that even if individual doses are missed, the drops still exert a therapeutic effect on the ocular surface.

The study by Daull et al. also demonstrated that intensive dosing (21 times daily) did not have negative effects on the cornea, which supports our approach [35].

During our study, we had several patients who, despite being instructed to use the drops 5 times daily for 7 days, were only applying them 2–3 times daily by the second follow-up visit. As a result, they were excluded from this study. This confirms that patients often do not adhere to recommendations, further justifying our approach to more intensive dosing to ensure the effectiveness of the treatment.

Based on these results, we believe that the seven-day period of using drops five times daily was appropriate and sufficient to achieve the desired clinical effects. However, in future studies, different dosing regimens could be considered to more precisely determine the optimal duration and frequency of drop use.

These gender differences may be attributed to hormonal differences affecting tear production and ocular surface health [36,37]. Such findings underscore the need for a personalized approach to treatment that takes into account gender-specific responses to preoperative DED management [36,37].

Patient outcomes post-cataract surgery are not limited to mitigating symptoms related to DED, but also include achieving superior visual acuity and the ability to view comfortably without glasses. The preoperative management of the ocular surface, such as through the application of lubricating eye drops, can contribute to minimizing unforeseen refractive errors and enhancing patient satisfaction. The research corroborates that dry eye syndrome can influence the precision of biometric measurements and keratometry, which are critical for the selection of the appropriate intraocular lens [38,39,40,41,42,43].

Postoperative DED is one of the primary causes of patient dissatisfaction following the implantation of extended depth-of-focus intraocular lenses (EDOF IOLs). In the studied cohort, 26.5% of eyes exhibited signs of DED, significantly contributing to complaints of blurred vision and photic disturbances, such as glare and halos [44].

Preoperative ocular surface optimization and careful lens selection can lead to better outcomes and greater patient satisfaction. This knowledge can inform the creation of specific preoperative protocols, such as thorough DED diagnostics and tailored moisturizing therapies, to minimize postoperative DED risk and enhance overall patient satisfaction.

In this study, we utilized 0.3% HPMC solution (Keratostill) eye drops, which comprise 0.3% HPMC, 5% dexpanthenol, disodium edetate, disodium phosphate dihydrate, potassium phosphate, purified water, and 0.01% cetrimide as a preservative. HPMC, a semi-synthetic cellulose polymer, is widely employed in pharmaceutical and cosmetic formulations due to its viscoelastic and protective properties.

The use of HPMC has been shown to significantly reduce the OSDI scores, indicating an improvement in dry eye symptoms [45]. By stabilizing the tear film, reducing evaporation, and increasing the tear film break-up time (TF-BUT), HPMC helps protect the ocular surface and supports overall ocular health [46].

In turn, the use of 2% HPMC during cataract surgery has been shown to significantly reduce postoperative dry eye symptoms, as demonstrated by a decrease in the OSDI scores [47]. These findings indicate that HPMC plays a protective role in preserving ocular surface health and minimizing discomfort following cataract surgery.

Dexpanthenol is an alcoholic analog of pantothenic acid, belonging to the group of B vitamins. A study conducted by Sabur and collaborators compared the efficacy of a combination of dexpanthenol and sodium hyaluronate with sodium hyaluronate alone in the treatment of DED and ocular surface inflammation after cataract surgery. The results showed that the combination of these ingredients was more effective in reducing dry eye symptoms and improving ocular surface inflammation than sodium hyaluronate drops alone [48]. Adding dexpanthenol significantly improved the healing process of the corneal epithelium after cross-linking procedures as well, accelerating re-epithelialization and subepithelial nerve regeneration, which surpassed the effectiveness of sodium hyaluronate drops alone [49]. Similar effects of dexpanthenol in combination with sodium hyaluronate were demonstrated in a study by Köppe and collaborators [50]. These properties of dexpanthenol make it a valuable ingredient for supporting hydration, epithelial regeneration, and reducing irritation, confirming its value in the treatment of the cornea after ophthalmic surgeries. Additionally, while our study employed OCT, TBUT, and the OSDI questionnaire to assess key clinical aspects of DED, we acknowledge that these three tests may not provide a comprehensive evaluation of the condition. As referenced by Tsubota et al. [51], Craig et al. [52], and Wolffsohn et al. [53], a more thorough assessment of DED could involve additional diagnostic tools and classifications, such as those recommended by the Asia Dry Eye Society and the TFOS DEWS II report. However, the tests selected for this study were appropriate for addressing our specific research objectives.

Despite the significant findings, this study has several limitations. One major limitation is the exclusive use of Keratostill moisturizing eye drops (0.3% HPMC) without comparing different artificial tear formulations or including a control group using sterile saline.

Naderi et al. emphasize that future research should focus on identifying the most effective preoperative interventions for DED in cataract surgery patients [9]. Our study highlights the need for further randomized controlled trials comparing different types of artificial tears and alternative treatment strategies.

This study has several limitations that should be acknowledged. First, the lack of a placebo group limits the ability to differentiate the specific effects of the active ingredients in the moisturizing drops from general lubrication effects. Additionally, the short follow-up period of only two weeks may not fully capture the long-term benefits or potential regression of the observed improvements. Future studies should consider extended follow-up durations to assess the sustainability of preoperative tear supplementation effects.

Second, while randomization helped reduce selection bias, it resulted in a significant baseline difference in the OSDI scores between groups, which may have influenced the outcome measures. Moreover, patient adherence to the preoperative eye drop regimen was self-reported, introducing the risk of underestimation or overestimation. Future studies should incorporate objective monitoring methods, such as electronic dose-tracking systems, to ensure an accurate compliance assessment.

Third, the diagnostic assessment of DED was based on a limited test battery, including the OSDI, TBUT, and anterior segment OCT. While these are commonly used in clinical practice, they do not meet the full diagnostic framework outlined by TFOS DEWS II guidelines. The absence of Schirmer’s test, tear osmolarity, meibography, or ocular surface staining may limit the diagnostic accuracy and restrict the interpretation of dry eye subtypes and severity. However, Schirmer’s test was intentionally excluded due to its low reliability and discomfort, especially in mild evaporative DED. Moreover, this study was not designed to assess aqueous-deficient subtypes, where Schirmer’s testing may have greater diagnostic value [54,55].

Additionally, the OSDI questionnaire, although widely validated, was modified in this study by excluding two vision-related items (“blurred vision” and “poor vision”) due to the universal presence of clinically significant cataracts in the study population. This adaptation was made to minimize the risk of confounding, as these items predominantly reflect cataract-induced visual impairment rather than ocular surface discomfort. The modified version was applied consistently across all participants and the scores were recalculated using the standard OSDI formula, adjusted for the number of completed items, thereby preserving the integrity of between-group comparisons.

It is important to acknowledge, however, that the modified OSDI was not formally revalidated. Although similar adaptations have been reported in the literature—including studies by Kasetsuwan et al., Palkovits et al., and Larmo et al.—the psychometric properties of our modified version may differ from those of the original instrument [22,56,57]. Moreover, since the aim of this study was not to diagnose dry eye disease, but to evaluate the subjective impact of artificial tear use on the postoperative ocular surface condition and patient comfort, the questionnaire served primarily as a practical symptom-assessment tool rather than a diagnostic measure. Nevertheless, the lack of a formal validation of the modified OSDI version remains a limitation and should be considered when interpreting the findings.

In terms of the procedural variables, although all surgeries were performed by the same experienced surgeon using a standardized phacoemulsification technique (stop-and-chop) under consistent lighting and anesthesia, individual ocular surface responses to operative stress, light exposure, and microenvironmental variations could not be entirely controlled. Preoperative measurements were conducted prior to any mydriatic or anesthetic instillation to avoid the distortion of the baseline tear film status, but external factors such as the room humidity, blinking patterns, and patient fixation may have introduced variability.

Each diagnostic method used also has inherent limitations. The OSDI is subjective and influenced by the patient perception, psychological state, and day-to-day variability. The TBUT, although widely used, is examiner-dependent and influenced by non-standardized fluorescein application, ambient conditions, and patient cooperation. OCT provides high-resolution epithelial mapping but does not assess the tear composition or inflammatory activity, and its measurements can be affected by the blink rate, tear film dynamics, and alignment accuracy.

In light of these considerations, future research should include placebo-controlled designs, larger and more balanced sample sizes, formally validated or unmodified questionnaires, and a broader diagnostic battery that may include tear osmolarity, meibomian gland imaging, in vivo confocal microscopy, and interferometry. These approaches would allow for a more comprehensive characterization of the ocular surface and the better elucidation of the effects of preoperative tear supplementation in cataract surgery patients.

## 5. Conclusions

This study demonstrates that the short-term preoperative use of moisturizing eye drops (Keratostill, 0.3% HPMC) may significantly improve tear film stability, ocular surface parameters, and patient-reported dry eye symptoms in individuals undergoing cataract surgery. Patients who received preoperative artificial tears showed significant improvements in their OSDI scores and TBUT (*p* < 0.001 and *p* = 0.002, respectively) compared to the control group. Preoperative use of lubricating eye drops appeared to protect the corneal epithelium, as only the control group showed a significant postoperative decrease in epithelial thickness (*p* = 0.021), while thickness was preserved in the test group.

However, these results reflect early postoperative outcomes over a limited two-week follow-up period, and the modified OSDI used in this study—although pragmatically justified—was not formally revalidated. Therefore, the findings should be interpreted with caution.

In summary, the results support the potential value of integrating short-term preoperative dry eye management into cataract surgery protocols to enhance early postoperative outcomes and patient comfort. Further research with validated diagnostic tools, longer-term follow-up, and comparisons across different tear formulations is needed to confirm these effects and define optimal treatment strategies.

## Figures and Tables

**Table 1:** Demographic characterization of the participants.

Age Group/Gender	Total (n = 71)	Control Group (n = 34)	Test Group (n = 37)
30–49	2	1	1
50–59	3	2	0
60–69	19	9	10
70–79	30	13	17
80–89	18	9	9
Female	44 (62%)	21 (61.8%)	23 (62.2%)
Male	27 (38%)	13 (28.2%)	14 (37.8%)

**Table 2:** Distributions of medical parameters studied by group at baseline, N = 71.

Parameter	Control (n = 34)	Test (n = 37)	*p*-Value
OSDI score	3.1 (0.0, 6.9)	12.5 (3.1, 21.4)	0.002
TBUT (s)	6.0 (4.3, 10.0)	5.0 (4.0, 9.0)	0.410
Corneal Epithelial Thickness (μm)	54.0 (52.0, 56.0)	53.0 (50.0, 57.0)	0.180

**Table 3:** EMMs of OSDI by group over time.

Group	Time	EMM	SE	95% CI (Lower Limit)	95% CI (Upper Limit)
Control	Baseline	3.92	1.13	1.71	6.13
Control	Preoperative	4.88	1.13	2.67	7.09
Control	Postoperative	3.70	1.13	1.49	5.91
Test	Baseline	11.81	1.11	9.63	13.98
Test	Preoperative	6.34	1.11	4.17	8.52
Test	Postoperative	3.30	1.11	1.12	5.47

95% Cl, confidence intervals; EMMs, estimated marginal means; OSDI, Ocular Surface Disease Index; SE, standard error.

**Table 4:** Contrast analysis of OSDI within time by the groups.

Group	Contrast	Estimate	SE	z	*p*	d
Control	Baseline–Preoperative	−0.96	0.65	−1.47	0.305	−0.16
Control	Baseline–Postoperative	0.22	0.65	0.34	0.938	0.04
Control	Preoperative–Postoperative	1.18	0.65	1.81	0.165	0.20
Test	Baseline–Preoperative	5.47	0.62	8.77	<0.001	0.94
Test	Baseline–Postoperative	8.51	0.62	13.65	<0.001	1.46
Test	Preoperative–Postoperative	3.04	0.62	4.88	<0.001	0.52

OSDI, Ocular Surface Disease Index; SE, standard error; z, z-score; *p*, *p*-value; d, effect size.

**Table 5:** Contrast analysis of OSDI between the groups at distinct time points.

Contrast	Time	Estimate	SE	z	*p*-Value	d
Control-Test	Baseline	−7.89	1.57	−5.03	<0.001	−1.36
Control-Test	Preoperative	−1.47	1.57	−0.93	0.350	−0.25
Control-Test	Postoperative	0.40	1.57	0.25	0.799	0.07

OSDI, Ocular Surface Disease Index; SE, standard error; z, z-score; d, effect size.

**Table 6:** Contrast analysis of corneal epithelial thickness at the middle control point within time by the eye status and groups.

Eye Status	Group	Contrast	Est.	SE	z	*p*	d
Non-operated	Control	Baseline–Preoperative	−0.05	0.54	−0.10	0.994	−0.01
Non-operated	Control	Baseline–Postoperative	−0.95	0.54	−1.76	0.182	−0.26
Non-operated	Control	Preoperative–Postoperative	−0.90	0.54	−1.66	0.220	−0.24
Non-operated	Test	Baseline–Preoperative	−0.93	0.52	−1.80	0.169	−0.25
Non-operated	Test	Baseline–Postoperative	−0.65	0.52	−1.26	0.419	−0.18
Non-operated	Test	Preoperative–Postoperative	0.28	0.52	0.54	0.850	0.08
Operated	Control	Baseline–Preoperative	0.56	0.54	1.03	0.555	0.15
Operated	Control	Baseline–Postoperative	1.45	0.54	2.67	0.021	0.39
Operated	Control	Preoperative–Postoperative	0.89	0.54	1.64	0.230	0.24
Operated	Test	Baseline–Preoperative	0.08	0.52	0.16	0.987	0.02
Operated	Test	Baseline–Postoperative	0.71	0.52	1.37	0.354	0.19
Operated	Test	Preoperative–Postoperative	0.63	0.52	1.22	0.442	0.17

Est., Estimate; SE, standard error; z, z-score; *p*, *p*-value; d, effect size.

**Table 7:** Contrast analysis of TBUT within time by eye status and groups.

Eye Status	Group	Contrast	Estimate (Est.)	Standard Error (SE)	z	*p*	Cohen’s d (d)
Non-Operated	Control	Baseline–Preoperative	0.20	0.53	0.38	0.922	0.13
Non-Operated	Control	Baseline–Postoperative	−0.59	0.53	−1.12	0.504	−0.38
Non-Operated	Control	Preoperative–Postoperative	−0.80	0.53	−1.50	0.291	-0.50
Non-Operated	Test	Baseline–Preoperative	−0.75	0.51	−1.47	0.304	−0.47
Non-Operated	Test	Baseline–Postoperative	−0.72	0.51	−1.41	0.336	−0.45
Non-Operated	Test	Preoperative–Postoperative	0.03	0.51	0.06	0.998	0.02
Operated	Control	Baseline–Preoperative	0.82	0.53	1.54	0.272	0.52
Operated	Control	Baseline–Postoperative	1.09	0.53	2.05	0.099	0.69
Operated	Control	Preoperative–Postoperative	0.27	0.53	0.51	0.864	0.17
Operated	Test	Baseline–Preoperative	−1.76	0.51	−3.46	0.002	−1.12
Operated	Test	Baseline–Postoperative	−1.58	0.51	−3.10	0.005	−1.00
Operated	Test	Preoperative–Postoperative	0.18	0.51	0.36	0.931	0.12

## Data Availability

The data used to support the findings of this study are included in the article. The data cannot be shared due to third-party rights and commercial confidentiality.
